# KI-67 as a predictive indicator of papillary thyroid cancer in Iraqi patients

**DOI:** 10.14341/probl13410

**Published:** 2024-03-07

**Authors:** Noor Mohammed Al-Timimi, Abed Hassan Baraaj

**Affiliations:** Uniuniversity of Baghdad; Uniuniversity of Baghdad

**Keywords:** Immuhohistochemistry, Iraqi patients, KI-67, MKI-67, papillary thyroid carcinoma

## Abstract

BACKGROUND. KI-67 (MKI-67 in humans) is a protein able to bind to DNA which contributes to cell growth and cell proliferation. KI-67 is currently considered as a biomarker that is widely utilized as prognostic indicator for evaluating cell proliferation, diagnosing diseases, and conducting research. Several different kinds of cancer have high Ki-67 expression, which simplifying the choice of treatment for individuals with various cancer types.AIM. The objective was to evaluate the expression of KI67 in patients suffering papillary thyroid cancer (PTC) also the association between patients age and gender and KI67 expression.MATERIALS AND METHODS. To undertake an in-depth investigation of KI67 in malignant and normal tissues, we used thyroid tissue sections to analyze KI67 expression in 70 samples, 50 different PTC (44 female and 6 male), and 20 normal types (10 for each gender). Each group’s average age is between 20 and 60.RESULTS. The analysis of the data revealed a substantial difference in the expression of ki67 between the patients and control groups. Ki67 expression and either gender or age did not significantly correlate.CONCLUSION. This study suggest that KI67 may be a crucial marker for assessing the aggressiveness of tumors and inflammatory diseases.

## RATIONALE

The thyroid malignance represent the most typical endocrine malignancy, affecting few than 1 to 2% of the cancer cases [1][2]. The histological characteristics enabled the categorization of thyroid cancer which involve anaplastic thyroid cancer, medullary thyroid cancer, poorly differentiated thyroid cancer, follicular thyroid cancer and papillary thyroid cancer [3].

Cancer in thyroid is usually diagnosed by several technique for example ultrasonography, magnetic resonance imaging (MRI), fine needle aspiration, computed tomography (CT) and radionuclide imaging. As stated by the patients reports throughout the diagnosis age, sex, lymph node, tumor size, metastases, and the cancer pathological differentiation are considered as prognosis risk factors [4]. Yet, no studies available that employ biomarkers to predict thyroid cancer prognosis. Proliferation of cells has the most important effect on the biological behavior of cancers. The marker Ki-67 is an antigen in nucleaus, which can performs in cell nuclei through active phases (G1, S, G2, and mitosis) excepting those in the quiescent cells phase (G0). These characteristics make the Ki-67 antigen a highly effective marker for the detection of cells that are rapidly proliferating in both normal and malignant cell populations.

Numerous techniques may be used to measure cellular proliferation, however immunohistochemical staining for the Ki-67 antigen is the one that is most often investigated. It is now frequently utilized in histology, notably as a proliferation marker in different tumor types. Overexpression of Ki-67 has been demonstrated to be related with neoplasms ranging from thyroid, breast, prostate, lung, bladder, kidney, as well as neuroendocrine tumors in major human investigations [5]. Ki-67 staining index, or the ratio of immunoreactive cell nuclei, has been utilized to direct treatment decisions in cases of hormone susceptible breast cancer, in addition to its usage in diagnostic and prognosis investigations of different neoplasms [6].

Despite this, only a few researchers investigated the link between Ki67 marker and thyroid cancer. The study of Lindfors et al. [7] studied the prediction utility of the Ki67 marker staining index in the papillary thyroid cancer which found that patients with PTC who would survive and not develop any illness had a different prognostic factor.Somuncu [8] found that the intensity of expression of Ki67 were higher in papillary thyroid cancer and microcarcinoma groups than in benign thyroid disease group. However, the aim of this research was to detect the Ki-67 expression levels in Iraqi patients with papillary thyroid carcinoma along the relationship between gender and age and this marker (KI-67).

## AIM OF THE STUDY

The aim was to evaluate the KI-67 biomarker expression in papillary thyroid carcinoma patients in Iraq as well as detection of the age and gender relation to this biomarker (ki67).

## MATERIALS AND METHODS

## Site and time of the study

The study took place between 2022 and 2023 at the biology department of the college of science at Baghdad University and the pathology department of the college of medicine at Al-Nahrain University. The thyroid tissue samples utilized in this study were paraffin blocks acquired from the laboratories of AlKindi Teaching Hospital, Teaching Laboratories of Medical City, Forensic Medicine of Medical City, and Alshariqa private laboratory between 2019 and 2022.

## Study populations (one or more)

In this study, 70 paraffin blocks containing thyroid tissues were employed, comprising 20 thyroid gland tissues used as negative controls (10 males and 10 females) and 50 papillary thyroid cancer (6 male and 44 female) to detect the Ki67 using immunohistochemistry approach. The ages of each group range from 20 to 60.

## Sampling method from the study population (or several sampling methods from several study populations)

Negative control and thyroid tissue samples were sliced to a thickness of 5 µm and put on positive charge slides for immunohistochemistry to determine the expression level of Ki-67 using the abcam data sheet.

## Study design

The design of this study involved the following:

## Statistical analysis

Then the data was analyzed by using SPSS program. Categorical variables were presented as numbers and percentages. The Chi-square test was performed to assess the connection between categorical variables, and any necessary changes were made. To compare the mean values of two groups the Mann Whitney U Test and the t -Test was used.

## Ethical review

The ethics committee of the Iraqi college of science at Baghdad University gave its approval to this research. (Number CSEC/0922/0095 in 26/September/2022).

## RESULTS

## KI-67 expression

The expression levels of KI-67 varies significantly (P≥0.001) between PTC patients and healthy controls. Depending on the level of expression, this marker’s intensity changed (Table 1). This marker was strongly expressed in 20 cases, or 40% of the patients (Figure 2 D), moderate expression was observed in 12 cases, or 24% of the total number of patients (Figure 2 C), and low expression was shown in 17 cases, or 23% of the patients (Figure 2 B). One instance, which represented 2% of patients had negative Ki-67 expression (Figure 1 B). All cases of the healthy controls representing 100% had negative Ki-67 expression (Table 1; Figure 1, 2 A).

**Table:** Table 1. Ki-67 scores between patients and healthy control * P value by Yates chi square test

Score		Patients N=50	Control N=20	P value
KI-67	High; N (%)	20 (40.0)	0 (0.0)	<0.001*
Intermediate; N (%)	12 (24.0)	0 (0.0)
Low; N (%)	17 (34.0)	0 (0.0)
Negative; N (%)	1 (2.0)	20 (100)

**Figure:**
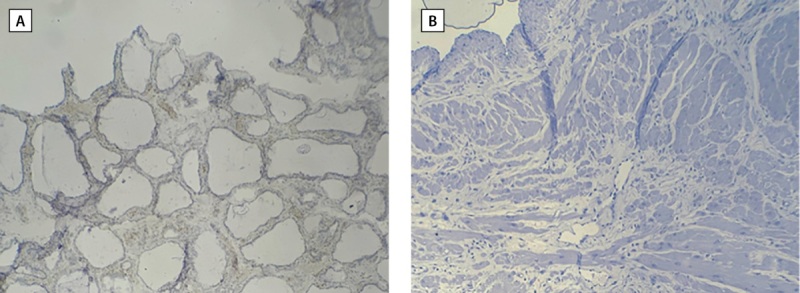
Figure 1. Cross section of thyroid tissue in (A) healthy tissue (B) malignant papillary thyroid tissue by using IHC that revealed negative expression of Ki 67 (less than 5%).10X

**Figure:**
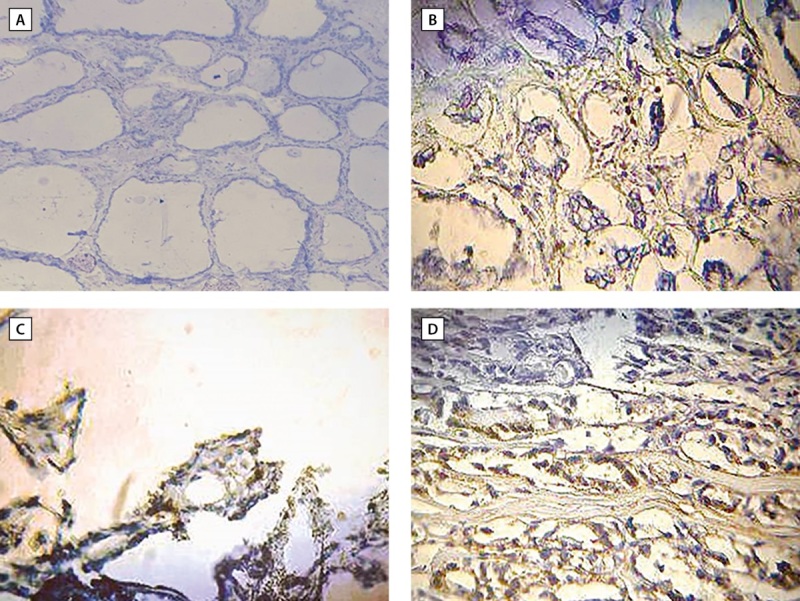
Figure 2. Cross section of thyroid tissue in (A) control showed negative expression of KI-67 (less than 5%). (B) Patient with PTC showed low expression of KI-67 (less than 5%). (C) Patient with PTC showed intermediate expression of KI-67 represent (5-10%). (D) Patient with PTC showed high expression of KI67 represent more than 10%. 40X

## KI-67 Expression in relation to the gender of patients

The gender of patients suffering from papillary thyroid malignant do not differ significantly in the expression level of KI-67 (Table 2). Females expressed this marker at different intensities, with high intensity in 20 cases, middle intensity in 11, low intensity in 12, and negative intensity in one case. Only two intensities of Ki-67 expression were present in males: moderate in one case and low in five cases (Table 2).

**Table:** Table 2. Scores of KI-67 expression according to sex in patients group * P value by Yates chi square test

Score		Male N=6	Female N=44	P value
KI-67	High; N (%)	0 (0.0)	20 (45.5)	0.092*
Intermediate; N (%)	1 (16.7)	11 (25)
Low; N (%)	5 (83.3)	12 (27.3)
Negative; N (%)	0 (0.0)	1 (2.3)

## Expression of KI-67 according to patients age

There was no discernible correlation between patient age and KI-67 expression (Table 3). Among those under the age of 25: high (3 cases) and moderate (2 cases). Ages 26 to 35 show high intensity (9 cases), intermediate (5 cases), and low intensity (6 cases).

**Table:** Table 3. Ki-67 scores according to age groups in patients group * P value by Yates chi square test

Score		Age (yr)	P value
≤25 N=5	26-35 N=20	36-45 N=17	>45 N=8
KI-67	High; N (%)	3 (60.0)	9 (45.0)	5 (29.4)	3 (37.5)	0.750*
Intermediate; N (%)	2 (40.0)	5 (25.0)	2 (11.8)	3 (37.5)
Low; N (%)	0 (0.0)	6 (30.0)	9 (52.9)	2 (25.0)
Negative; N (%)	0 (0.0)	0 (0.0)	1 (5.9)	0 (0.0)

Patients between the ages of 36 and 45 have showed the marker at all levels of intensity. One case was negative, nine had low expression, two had moderate expression, and five had high expression. Over 45 patients had the marker expressed, with three having strong expression, three having moderate expression, and two having low expression (Table 3).

## DISCUSSION

The biological activity of malignant cells is assumed to be largely influenced by cell proliferation, tumor genesis is considered critically impacted when there is an imbalance between apoptosis and cell proliferation. The proliferation of cell may be measured via the IHC method, which is used to identify an antigens specific to cycle. The tumor cell ability to proliferate can be determined by the biomarker Ki-67/MIB-1 and the high level of this biomarker is related to metastasis in numerous malignancies [9].

## Expression of KI-67

The current finding reveled a significant difference in the expression value of KI-67 between patients with PTC and healthy controls. The strength of this marker varied with the degree of expression. In patients with PTC, the KI-67 expression relevance may be partially explained by its biological mechanism. Ki-67, a well-known biomarker of cell proliferation in various cancers [10][11][12], is essential for mitosis because it controls chromatin recombinant.

Ki67 is a protein bonded to DNA present in the nucleus, which is linked to growth of cell. This marker has been greater utilized in medications and researches of numerous types of cancer as its one of the key evidence of cell proliferation. The principle function of this marker is to protect the structure of DNA throughout cell division. In cell division process the Ki67 protein is phosphorylated and dephosphorylated, and it is also subjected to proteases and monitored by proteolytic pathways [13]. In addition, the Ki67 structure is resembles to that of certain proteins that regulate the cell cycle and is lost in quiescent cells (G0), but it begins to emerge in the nucleus during the G1 stage [14]. Then, throughout the S and G2 stages of cell cycle, Ki67 protein levels progressively rises reaching the top during the M phase, before rapidly declining in the late M phase [14]. Because of the direct linkage between the expressions of Ki67 marker and tumor cell proliferation or growth, therefore, this marker is used as a marker in routine pathological research. According to research by Davey et al. [10] and Maia et al. [11] found that the greater Ki-67 expression is typically associated to bad prognosis in cases of breast and prostate cancer. However, just a few studies have examined Ki67 in thyroid where there it was cancer or disease. Ito et al [7] investigated the prognostic relevance of a Ki-67 staining index in papillary thyroid cancer and demonstrated that Ki67 was a predictive factor of PTC patients for disease-free survival.

The current results are in line with a study by [8], which found that the PTC group’s Ki67 expression density was much greater than the group with benign thyroid illness. Another study that supported our findings showed that the thyroid tissues with benign papillary hyperplasia (BPH) and PTC had significantly different levels of Ki67 expression [15].

## Expression of KI-67 according to patient’s gender

The expression level of Ki-67 in patients with PTC does not differ significantly across sexes and different intensities of this marker were expressed in female than in male. This difference in KI67 expression between males and females may be related to environmental, menstrual, and reproductive variables. The current result is consistent with a study by [16] that revealed women are more likely than men to develop papillary thyroid cancer.

## Expression of KI-67 according to patient’s ages

The current finding found that the age of patient did not appear to be correlated with Ki-67 expression. This could suggest that Ki-67 is used to predict the prognosis of any tumor. Ki-67 may be found in all proliferating cells and serves as a proliferation marker. One of the risk factors for the prognosis of thyroid cancer is age upon diagnosis [17].

Lei et al [18] found that Ki-67 mRNA expression levels in PTC patients were higher in the older age group (55) than in the younger age group (55).This could be related to the deficiency of immune system in older patient. Whereas, Siironen et al [19] observed that Ki-67 expression considerably rises with aging, suggesting that tumors in older patients may develop more quickly. This increased proliferative activity might be explain the worse prognosis in these patients.

The present results concur with those of Zhou et al [15] who observed no significant correlation between genders, age, or the intensity of Ki67 expression and positive expression rates in PTMC. Deng-hua Pan et al [20] disagree with the present finding, who found statistical correlation between Ki-67 and age (odds ratio =1.71, 95% confidence interval: 1.14-2.57, P=0.010).

## CONCLUSION

Today, the most significant indicator of cell proliferative activity is Ki-67, which has been shown to be a valid predictor of tumor malignancy, prognosis, and course of treatment. The group of patients with papillary thyroid cancer is varied in terms of the biological characteristics of the tumor and, consequently, the prognosis. Therefore, the topic has been little studied, although there is a significant need for it.

The expression of Ki-67 was found to be significant between the patients and the healthy group in the current investigation, and it was not found to be influenced by the age or gender of the patients undergoing compression. To further corroborate our findings, however, carefully planned prospective studies are needed.

## ADDITIONAL INFORMATION

Funding. No funding

Conflict of interest. The authors declare no obvious and potential conflicts of interest related to the content of this article

Contribution of authors. Before publishing, the manuscript was reviewed and authorized by all writers, who also acknowledged that they were accountable for every component of the work, which implied that any concerns about the accuracy or integrity of any portion of the work would be properly examined and resolved.

Acknowledgment. For those who helped me with any kind of encouragement, warmly advice or knowledge and where the sparkling light through the dark days epically Dr. Genan Adnan Al-bairuty, Dr. Mohammed Naji Taha, Dr. Khalid Waleed Qassim and Dr. Atta Awad Al-timimi.
